# Cilia develop long-lasting contacts, with other cilia

**DOI:** 10.1186/2046-2530-1-5

**Published:** 2012-05-04

**Authors:** Peter K Jackson

**Affiliations:** 1 Genentech Inc, South San Francisco, CA, 94080, USA

## 

The primary cilium has been much celebrated for its central role as a sensory organelle. Whether we consider odorants binding to odorant receptors in olfactory cilia or light stimulating rhodopsin in the retinal outer segment, core ciliary structures and G-protein coupled receptors like rhodopsin allow our senses to function. The importance of ciliary pathways in sensation is well illustrated in patients with Bardet-Biedl syndrome, whose ciliary defects are linked to anosmia and loss of retinal function [1]. In addition to sensation, other ciliary GPCRs may bind specific hormones, like serotonin or somatostatin, to control physiological signals. Other physical means of ciliary signalling have been considered, including flow or mechanosensory sensing, binding of exosomes, and even lateral contacts with supporting cells for neurons in chordotonal organs. But can cilia reach out and touch one another? A report from the Lippincott-Schwartz lab, in the launch issue of *Cilia,* suggests that not only do cilia touch, they may even stick together for a long time [2].

Cilia are organized around the axoneme, with the centriole organizing the ciliary base [see [1,3], for reviews]. Around the axoneme, the ciliary membrane forms an extended sheath, organizing ciliary receptors and other membrane proteins. At the base of the cilium, the membrane typically shows an infolding or ciliary pocket, which can be a site for endocytosis. At the distal end of the cilium, the ciliary tip is a specialized segment of membrane that extends from the axoneme, and may serve a specialized function in signalling. The ciliary tip may also be a site where exosomes pinch off. A scanning electron microscopy study from the LaRusso lab observed exosomes in close apposition to cholangiocytes in ciliated bile ducts, and showed that isolated exosomes containing polycystin-1, -2, and fibrocystin can efficiently bind to cholangiocyte cilia [4,5]. The study suggested that membrane bound structures can associate with the ciliary membrane, and that specific paracrine effects of exosomes might be mediated by binding to cilia. A study from the Kernan lab in chordotonal organs of *Drosophila* suggested that a specialized ciliary diliation, a narrowing of the cilia, was organized by the intraflagellar transport (IFT-A) complex, and this ciliary specialization interacted with supporting cells in the chordotonal organ and was required for sensory function [6]. Finally, a number of studies show that the distal end of the retinal outer segment is in close juxtaposition to invaginations in retinal pigment epithelial cells (RPE), and that sloughing of outer segments triggers local phagocytosis by the RPE [7]. Finally, in *Chylamydomonas* induced to mating, the cilia can form agglutination structures critical to their mating function [8].

So cilia appear to interact purposefully with cells and exosomes from their surrounding cellular neighbourhood. But what about touching each other?

Lippincott-Schwartz and colleagues consider cilia-cilia pairing, first looking into the retina and liver. In the retina, they observe that the ciliary membrane of rod cells, enveloping both the connecting cilium and outer segment, are closely packed side-by- side in the photoreceptor layer. The close juxtaposition of outer segments suggested possible adhesion sites. In the liver cholangiocytes, cilia extend into the bile duct and may participate in bile flow and signalling. Some cilia appeared to participate in cilia-cilia contacts, especially in younger animals. Moving to a cell culture model, the authors find that when MDCK cells are induced to form 3D cysts, the cilia are oriented on the apical surface towards the lumen, and indeed form cilia-cilia contacts. In MDCK cell monolayers grown on filters, the cilia are longer (815) and cells are close packed (10 spacing). Here the cilia can form continuous arcs, or point contacts, and even cilia-cilia networks. In other cells, less tightly packed and with short cilia, the cilia-cilia contacts are less apparent.

If the cilia-cilia contacts are important, a first guess is that they would be enduring, rather than transient. Indeed, the authors find that by filming of cells, that most showed contacts persisting for 2 hours, and over half of cell for up to 20 hours or longer. So cilia-cilia contacts can be stable; a first estimate is that 13% of cilia make contact. What sets the percentage and whether there are cell mechanisms that favor ciliary pairing would help us understand when and where the phenomena is important. One might guess that the density and organization of cells would be important to favor ciliary pairing. Some data suggest that ciliary length may be regulated once preciliated cells reach confluence and establish adherens junctions, possibly requiring the Par3-Par6-aPKC pathway [9,10]. We could consider a contact-dependent, ciliary extension model, where cells establish cell-cell contacts, completing the formation of adherens junctions or tight junctions, which would then trigger ciliary extension to allow cilia to reach out and establish cilia-cilia communication. An intriguing possibility is suggested by molecular properties of the NPHP1-4-8 network of nephrocystins, a group of genes defective in the nephronophthisis, a paediatric ciliopathy with renal insufficiency. The NPHP1-4-8 proteins have roles in both cell-cell and tight junction formation, and in the formation of the ciliary transition zone [11,12]. These proteins would be interesting candidates to communicate between cell-cell junctions and the cilium.

In terms of how the ciliary arcs are formed, the authors consider whether a single cilium might bend into an arc; or if cilia from adjacent cells adhere; or if cilia from adjacent cells actually fuse! Using time-lapse microscopy, it became clear that ciliary contacts are established first, and then zipped up to form a stable pairing. Electron micrographs of the interaction was reminiscent of an adhesive pairing, so the authors considered whether known molecular pathways for adhesion were required. Using EGTA to chelate calcium, and DTT to dissolve disulphide bonds, neither disrupted ciliary pairing, suggesting that neither calcium-dependent cadherins nor disulphide-requiring Ig domains were strongly required. In contrast, treating cells with the mannosidase II inhibitor swainsonine to block oligosaccharide formation, the authors did observe a loss of the ciliary pairing phenomena. Other drugs perturbing glycoprotein formation like neuraminidase treatment or elastase treated failed to block pairing, but these experiments were technically less conclusive. Considering the ever-important cell cycle, it was clear that as cilated cells enter mitosis, they also showed a loss of ciliary pairing, further supporting the potential biological relevance of the pairing phenomena. Because *Chylamydomonas* also uses glycoproteins for ciliary pairing, there is now question of which ciliary membrane protein(s) may mediate pairing. Inspection of the ciliary or FABB proteome may be a good place to look for candidates [13].

The studies here provide a provocative first glimpse of how glycoprotein contacts may link cilia-cilia pairings in cells, which could proceed from initial synaptic pairing to more stable, zipped-up associations. Additional genetic or molecular studies could inform and support the importance of these pairing for ciliary function. But the larger question is why cilia would use such contacts. Among the potential roles, a general function in intercellular communication between closely apposed cells or intracellular communication in tissues would be important. A somewhat similar mechanism may be provided by cytonemes, specific filopodial intercellular links [14], and much remains to be understood about the various physical connections that allow paracrine signalling in tissues. Indeed, aside from secreted factors, cilia could serve as a second important physical means to have cells communicate. Because ciliary signalling presumably predates neural signalling in evolution, we can imagine the intraciliary contacts would form to regulate a primitive synaptic system communicating local exocytic or cilia-bound signals to bind and trigger signalling to cilia on nearby cells. In highly organized structures like the retina, these cilia-cilia contacts may be important to also serve structural or architectural roles. It may be surprising that new ultrastructure can be discovered today, but as the present study illustrates, we first have to open our minds to the possibility of new modes of cell-cell communication, and then start to look for the evidence that informs us of how these pathways work. With the excitement around cilia, we are being continually surprised. Maybe some labs already have the sense that cilia-cilia pairings directly mediate cellular communication and can now see a path to publishing that data to help the rest of us.

## References

[B1] Baker K Beales PL Making sense of cilia in disease: the human ciliopathies Am J Med Genet C Semin Med Genet 2009 151C 4 281 295 19876933 10.1002/ajmg.c.30231

[B2] Ott C Elia N Jeong SY Insinna C Sengupta P Lippincott-Schwartz J Primary cilia utilize glycoprotein-dependent adhesion mechanisms to stabilize long-lasting cilia-cilia contacts Cilia 2012 1 3 10.1186/2046-2530-1-3 PMC3541541 23351752

[B3] Oh EC Katsanis N Cilia in vertebrate development and disease Development 2012 139 3 443 448 22223675 10.1242/dev.050054 PMC3275291

[B4] Huang BQ Ward CJ Masyuk AI Masyuk TV Harris PC LaRusso NF Exosome-like Vesicles are Present in the Lumen of Rat Bile Ducts and Interact with Cholangiocyte Primary Cilia Microsc Microanal 2007 13 258 259

[B5] Masyuk AI Huang BQ Ward CJ Gradilone SA Banales JM Masyuk TV Radtke B Splinter PL LaRusso NF Biliary exosomes influence cholangiocyte regulatory mechanisms and proliferation through interaction with primary cilia Am J Physiol Gastrointest Liver Physiol 2010 299 G990 G999 20634433 10.1152/ajpgi.00093.2010 PMC2957333

[B6] Lee E Sivan-Loukianova E Eberl DF Kernan MJ An IFT-A protein is required to delimit functionally distinct zones in mechanosensory cilia Curr Biol 2008 18 1899 1906 19097904 10.1016/j.cub.2008.11.020 PMC2615538

[B7] Insinna C Besharse JC Intraflagellar transport and the sensory outer segment of vertebrate photoreceptors Dev Dyn 2008 237 8 1982 1992 18489002 10.1002/dvdy.21554 PMC2692564

[B8] Pan J Snell WJ Kinesin-II is required for flagellar sensory transduction during fertilization in Chlamydomonas Mol Biol Cell 2004 13 1417 1426 11950949 10.1091/mbc.01-11-0531 PMC102279

[B9] Zhu D Shi S Wang H Liao K Growth arrest induces primary-cilium formation and sensitizes IGF-1-receptor signaling during differentiation induction of 3 T3-L1 preadipocytes J Cell Sci 2009 122 2760 2768 19596798 10.1242/jcs.046276

[B10] Sfakianos J Togawa A Maday S Hull M Pypaert M Cantley L Toomre D Mellman I Par3 functions in the biogenesis of the primary cilium in polarized epithelial cells J Cell Biol 2007 179 1133 1140 18070914 10.1083/jcb.200709111 PMC2140027

[B11] Delous M Hellman NE Gaude HM Silbermann F Le Bivic A Salomon R Antignac C Saunier S Nephrocystin-1 and nephrocystin-4 are required for epithelial morphogenesis and associate with PALS1/PATJ and Par6 Hum Mol Genet 2009 18 4711 4723 19755384 10.1093/hmg/ddp434 PMC2778369

[B12] Sang L Miller JJ Corbit KC Giles RH Brauer MJ Otto EA Baye LM Wen X Scales SJ Kwong M Huntzicker EG Sfakianos MK Sandoval W Bazan JF Kulkarni P Garcia-Gonzalo FR Seol AD O'Toole JF Held S Reutter HM Lane WS Rafiq MA Noor A Ansar M Devi AR Sheffield VC Slusarski DC Vincent JB Doherty DA Hildebrandt F Reiter JF Jackson PK Mapping the NPHP-JBTS-MKS Protein Network Reveals Ciliopathy Disease Genes and Pathways Cell 2011 145 513 528 21565611 10.1016/j.cell.2011.04.019 PMC3383065

[B13] Li JB Gerdes JM Haycraft CJ Fan Y Teslovich TM May-Simera H Li H Blacque OE Li L Leitch CC Lewis RA Green JS Parfrey PS Leroux MR Davidson WS Beales PL Guay-Woodford LM Yoder BK Stormo GD Katsanis N Dutcher SK Comparative genomics identifies a flagellar and basal body proteome that includes the BBS5 human disease gene Cell 2004 117 541 552 15137946 10.1016/s0092-8674(04)00450-7

[B14] Roy S Hsiung F Kornberg TB Specificity of Drosophila cytonemes for distinct signaling pathways Science 2011 332 354 358 21493861 10.1126/science.1198949 PMC3109072

